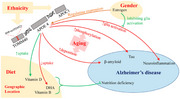# Genetics and Environmental Determinants of Apolipoprotein E‐linked Dementia in Older Africans

**DOI:** 10.1002/alz70855_107502

**Published:** 2025-12-24

**Authors:** Tobi Nifemi Olajide, Oluwatimilehin Oladapo, Chukwuebuka Stanley Asogwa, Gideon Olajide, Ayomide Oyekan, Ayomide Fatola, Timileyin Olanrewaju, Damola Oyegbile, Ikechukwu Ugbo, Henry Demian Oyoyo, Ridwan Kamarudeen, Olawale Famakin

**Affiliations:** ^1^ College Research and Innovation Hub, Ibadan, Nigeria; ^2^ College of Medicine University of Ibadan, Ibadan, Nigeria

## Abstract

**Background:**

Dementia is a growing public health concern in Africa, driven by an aging population and unique genetic and environmental factors. The Apolipoprotein E (APOE) ε4 allele, a well‐established genetic risk factor for Alzheimer's disease, has a nuanced role in African populations. This review highlights key findings, including evidence that the impact of APOE ε4 on dementia risk in Africa is attenuated compared to Western populations.

**Method:**

A systematic research and screening process was conducted to identify relevant literature on the research topic across electronic databases including PubMed/MEDLINE, African Journals Online and Google Scholar. Relevant data were then extracted from papers that met the inclusion criteria on the genetic and environmental determinants of APOE‐linked Dementia in older persons in Africa.

**Result:**

The impact of APOE ε4 on dementia risk in Africa is attenuated compared to Western populations. This attenuation may stem from genetic diversity, environmental interactions, and evolutionary adaptations, such as APOE ε4's protective effects against infectious diseases like malaria. Gene‐environment interactions, particularly in relation to education, environmental toxins, and air pollution, play a significant role in dementia risk in Africa. Educational attainment is associated with cognitive reserve, which may delay dementia onset, as seen in studies from Nigeria, Tanzania, South Africa, and Ghana. Additionally, environmental exposures like heavy metals and pesticides can contribute to cognitive decline, as seen with the neurotoxic effects of substances like DDT. Vitamin D deficiency, linked to insufficient sunlight exposure, also emerges as a potential risk factor for dementia. Furthermore, air pollution, particularly PM2.5 exposure, exacerbates existing dementia risk factors such as stroke, hypertension, and diabetes. Despite these insights, disparities in healthcare access, diagnostic practices, and the absence of culturally sensitive diagnostic tools hinder the accurate characterization of dementia phenotypes and APOE's role in African populations.

**Conclusion:**

This review emphasizes the need for targeted genetic research, improved healthcare infrastructure, and culturally tailored interventions. Collaborative initiatives like the African Dementia Consortium provide a vital foundation for addressing these challenges, improving dementia care, and advancing global understanding of ADRD in African contexts.